# Pedigree-Based Estimation of Y-STR Mutation and Male Differentiation Rates: Application to Historical Remains Identification

**DOI:** 10.3390/genes16101211

**Published:** 2025-10-14

**Authors:** Jasmine R. Connell, Toni White, Thais Zielke, Luke Armstrong, Natasha Mitchell, Lyn R. Griffiths

**Affiliations:** 1 Centre for Genomics and Personalised Health, Genomics Research Centre, School of Biomedical Sciences, Queensland University of Technology (QUT), 60 Musk Avenue, Brisbane, QLD 4059, Australia; 2 Unrecovered War Casualties—Army, Royal Australian Army, P.O. Box 7902, Canberra, ACT 2610, Australia

**Keywords:** male differentiation, historical remains, human identification, Y-STR, mutation rate, Yfiler Plus

## Abstract

Background/Objectives: High differentiation rates provided by Y-chromosomal short tandem repeats (Y-STRs) are highly advantageous in most forensic and genealogical casework, as they enhance the ability to exclude close or moderately related individuals, refine an individual’s position within a pedigree, and uncover the population substructure in otherwise homogeneous groups. However, the impact for historical remains identification casework is underexplored. Methods: We present a pedigree analysis of 366 males from 183 pedigrees, separated by 4 to 16 meioses at 27 Y-STR loci, from the Yfiler Plus kit. The differentiation rate for a given degree of separation was defined as the proportion of pairs at that specific number of meioses showing at least one allelic difference, relative to the total number of such pairs. Results: Our pedigree-based locus-specific mutation rates were consistent with published father–son values for 22 of 25 loci, with 3 loci (DYS389II, DYS449, and DYS570) being significantly different (*p* < 0.05). These results were consistent with previous pedigree-based estimates, and the strong agreement between father–son and pedigree-based mutation rates supports the use of pedigrees as a reliable method for estimating mutation rates. The probability of differentiating male relatives reached 60.1%, which is similar to previous studies using the Yfiler Plus kit. Conclusions: This high male differentiation rate is advantageous for distinguishing unrelated individuals within the same population, reducing false inclusions. However, when comparing distantly related individuals, excessive mutations accumulated over many generations may obscure genuine patrilineal relationships, increasing the risk of false exclusions. Our findings are likely to be highly valuable for future interpretation of Y-STR haplotypes from patrilineal relatives across a wide range of applications, with significant relevance to historical remains identification casework.

## 1. Introduction

Y-chromosome short tandem repeats (Y-STRs) have demonstrated significant utility in various fields such as forensic genetics, molecular anthropology, and genealogical studies because of their strict paternal inheritance and high discriminatory potential among unrelated males [1,2,3]. Y-STRs are located within the non-recombining region of the Y chromosome, and, because of this, are passed down through paternal lineages essentially unchanged, with the only source of variation between generations being mutation [4,5,6]. In forensic casework, Y-STR haplotypes are especially valuable in excluding male suspects as contributors to crime scene material, while matches have traditionally been evaluated through reference to population databases such as the Y Chromosome Haplotype Reference Database (YHRD; [7]). The increasing inclusion of rapidly mutating (RM) Y-STRs in commercial kits, such as Yfiler Plus, has markedly enhanced the discriminatory capacity of Y-STRs, not only between unrelated individuals but also among close paternal relatives [8,9,10].

Mutation rates are a critical parameter in the interpretation of Y-STR profiles, influencing both differentiation between paternal relatives and the statistical weight that can be attached to haplotype comparisons. RM loci exhibit mutation rates up to an order of magnitude higher than conventional markers, increasing the ability to distinguish closely related males and thereby improving outcomes in complex forensic cases and familial searching [4,5,6]. Hence, these markers are especially beneficial in scenarios involving missing persons, or mass disasters, where RM Y-STRs reduce the likelihood of coincidental matches and can offer finer resolution and individual identification [3,11,12,13,14]. However, this increased mutation rate can present interpretative challenges, particularly when comparing profiles from distantly related individuals. Over many generations, these mutations accumulate, meaning that even true paternal relatives may exhibit increased allelic mismatches. A key challenge addressed in this manuscript is understanding how many differences one might expect between two distantly related individuals, as is often encountered in historical forensic identification.

Early estimates of Y-STR mutation rates were almost exclusively based on close relatives, particularly father–son pairs, where the number of separating meioses is known with certainty and paternity can be confirmed with autosomal markers [15,16,17,18]. While this design ensures high confidence in observed mutational events, such studies require very large numbers of father–son pairs to achieve sufficient statistical power, and are often constrained by sample availability, cost, and labour. Pedigree-based studies offer a complementary approach by leveraging deep-rooted family structures in which many meioses can be represented with relatively few sampled males. This allows the accumulation of a larger number of meioses overall, theoretically increasing the precision of mutation rate estimates [19]. However, pedigree-based approaches also carry inherent challenges, including uncertainties from parallel or back mutations, multi-step changes, and misreported familial relationships [5,20,21]. Despite these limitations, recent studies have demonstrated concordance between pedigree-based and father–son-derived estimates, supporting the validity of pedigrees as a framework for mutation rate estimation [19]. Other studies have also extended Y-STR mutation analyses beyond close paternal relationships, examining individuals separated by up to four meioses [22] and, in a more limited sample, up to twenty meioses [4]. These investigations suggest that Y-STR profiles exhibiting two or fewer mutational differences are typically consistent with shared paternal ancestry, whereas higher levels of divergence are more indicative of unrelated male lineages. However, these studies employed earlier-generation Y-STR panels, and analysis was confined to relatively close relatives (≤4 meioses) or skewed towards lower degrees of separation (≤13 meioses).

Although previous investigations have highlighted the enhanced discriminatory power of RM Y-STRs (e.g., [4]), few have evaluated their cumulative mutational dynamics across extended paternal genealogies. Consequently, the interpretation of Y-STR mismatches in the context of distant paternal relationships, such as those encountered in historical human identification efforts, remains an unresolved and critical issue within forensic genetics. In Unrecovered War Casualties—Army (UWC-A) casework, skeletal remains of missing servicemembers are compared against DNA from distant paternal relatives (up to 16 meioses apart [23]), and, as the conflicts in question recede further into the past, the generational gap between potential family reference samples will continue to increase. Assumptions of haplotype concordance can be compromised by mutation events, and therefore high-resolution mutation data are particularly relevant in applied contexts such as the identification of historical or missing persons, where reference DNA samples often come from distant paternal relatives. In such cases, understanding the expected degree of differentiation between male relatives separated by multiple meioses is essential to avoid both false exclusions and overestimation of evidential value. While Ralf et al. [19] reported an analysis of male relative differentiation in distant relatives, additional studies remain limited.

In this study, we investigated Y-STR mutation and male relative differentiation rates using the Yfiler Plus kit across 183 pedigrees, encompassing 366 relatives separated by 4 to 16 meioses. By comparing our estimates with previously published father–son and pedigree-based studies, we aimed to evaluate the utility of pedigrees for mutation rate estimation, assess the discriminatory power of Yfiler Plus loci, and explore the implications of high differentiation rates for forensic casework and historical remains identification.

## 2. Materials and Methods

### 2.1. Ethics

This project received ethical approval from Queensland University of Technology and the Department of Defence Human Research Ethics Committees (2021000378 and 328-21 respectively). All analyses were performed in accordance with relevant guidelines and regulations, and all participants provided informed consent for research involvement. The study was conducted in accordance with the Declaration of Helsinki.

### 2.2. Sample Collection and Preparation

From September 2021 to December 2024, 674 male DNA samples were analysed, and, of these, 20 were excluded because they showed too much variation (i.e., more than 15 mutations) compared to other pedigree members to be reasonably considered paternally related. Another 8 samples were excluded from further analysis because of incomplete genotype data, and 280 were excluded because of the lack of another paternally related member with available genomic data. The remaining 366 males were from 183 pedigrees, separated by 4 to 16 meioses. The genealogical process for recruitment has been described previously [23]. These individuals were drawn from extended family pedigrees where at least two distantly related male descendants were available for Y-STR analysis. In each case, genealogical research indicated the relationship (e.g., 4 meioses could correspond to first cousins or a great-uncle–grand-nephew pair, whereas 16 meioses corresponds to seventh cousins under a symmetric pedigree; ‘once-removed’ relationships add one meiosis per generation offset).

Participants were provided with either a SpeciMAX Stabilised Saliva Collection Kit (Thermo Fisher Scientific, Waltham, MA, USA) or a PAXgene Saliva Collector (Qiagen, Hilden, Germany), which they returned to our laboratory upon collection. DNA was extracted from each saliva kit using prepIT.L2P reagent (DNA Genotek, Ottawa, ON, Canada) according to the manufacturer’s instructions. The quality and quantity of DNA were determined using the NanoDrop Spectrophotometer (Thermo Fisher Scientific) and Qubit dsDNA Quantification Assay (Invitrogen, Waltham, MA, USA).

### 2.3. Y-STR Genotyping

Samples were genotyped in a single multiplex reaction using the Yfiler Plus PCR Amplification Kit (Applied Biosystems, Waltham, MA, USA) as described previously [23]. This kit includes 27 Y-STR loci widely used in forensic laboratories and encompasses the traditional core Y-STRs with moderate mutation rates as well as several RM markers that have higher per-generation mutation frequencies. PCR products were separated and detected by capillary electrophoresis on an Applied Biosystems 3500 Genetic Analyser with POP-7 polymer (Applied Biosystems) as described previously [23]. Data analysis was performed using the software GeneMapper v6 (Applied Biosystems). All electropherograms and allele calls were carefully reviewed by a second independent analyst to confirm any cases of intermediate variants (e.g., duplicated alleles or null alleles indicating a deletion or primer-binding mutation).

### 2.4. Mutation Rate Estimation

To estimate Y-STR mutation rates using pedigree data, we adopted a frequentist framework similar to that described by Ralf et al. [19]. Mutation rates (μ) were estimated at each locus using the formula μ = x/N, where x is the number of observed mutations, and N is the total number of meioses. Importantly, in our study, each pedigree consisted of only two individuals; therefore, the number of pairwise meioses and actual meioses were equivalent, eliminating the risk of double-counting transmission events. This analysis was performed individually for each pedigree, after which, mutation counts and meioses were summed, allowing the per-marker rates to be estimated. Confidence intervals (CIs: 95%) were calculated using Epitools, an online tool provided by AusVet Animal Health Services [24]. The programme outputs intervals using five alternative calculation methods, as described by Brown et al. [25]. The Wilson and Clopper–Pearson methods were reported.

When estimating mutations from extended pedigrees (rather than direct father–son comparisons), several assumptions were necessary due to generational gaps or missing data:

1. When two males in a lineage showed no haplotypic differences, we assumed that no mutations occurred along those missing links because no intermediate relatives were available with genotype data.
2. Our analysis used extended pedigrees with no typed father–son pairs, and therefore the meiosis in which a mutation occurred could not be identified. Multi-step differences between patrilineal individuals were interpreted as multiple single-step mutations that were distributed along the connecting lineage. This rule applies only to non-adjacent relatives; in father–son pairs, a genuine multi-step event would be accepted as the only valid scenario.
3. Across all pedigrees, we applied a parsimony principle, always favouring the minimum number of mutational steps required to explain observed haplotypic differences.

These assumptions are expected to hold true for most cases, but we acknowledge that they may lead to errors in some. For example, genuine multi-step single-meiosis events may exist between non-adjacent relatives, and therefore these assumptions may slightly overcount the number of mutations occurring. However, we expect the impact on aggregate rate estimates is small.

For single-copy Y-STR loci, mutation inferences were straightforward. For example, a shift from allele 10 in one individual to allele 12 in another indicated two single-step mutations (as per Assumption 2). However, for multi-copy loci, more complex interpretations were required. In all scenarios, the guiding principle was to minimise inferred mutational steps while considering biological plausibility and data limitations. Statistical significance was determined using Fisher’s exact method.

### 2.5. Differentiation Rate Estimation

To estimate the Y-STR-based male relative differentiation rate across varying degrees of patrilineal separation, we again applied a frequentist approach following the methodology described by Ralf et al. [19]. Differentiation rates were calculated for relative pairs separated by 4 to 16 meioses. For each pair, we assessed whether at least one allelic difference was present across the tested Y-STR loci. The differentiation rate for a given degree of separation was defined as the proportion of pairs (at that specific number of meioses) showing at least one allelic difference, relative to the total number of such pairs. To quantify the statistical uncertainty of the estimates, 95% confidence intervals were calculated using the Clopper–Pearson method.

## 3. Results and Discussion

### 3.1. Summary Statistics

**Figure 1:**
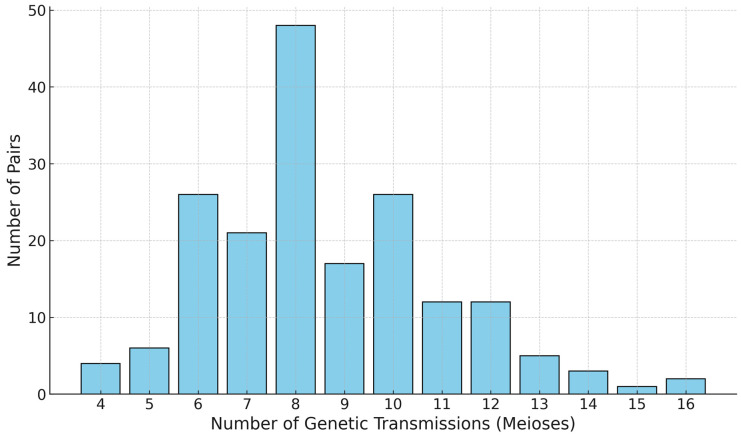
Breakdown of the paternally related pairs separated by 4 to 16 meioses (n = 183).

A total of 366 males from 183 pedigrees were analysed in this study, allowing the comparison of 183 paternally related pairs, separated by 4 to 16 meioses. Figure 1 provides a breakdown of the distribution of pairs. To determine the discrimination capacity, we took one individual from each unrelated family and observed 183 unique haplotypes among 183 unrelated men, which resulted in a haplotype discrimination capacity of 100%. This is consistent with previous studies [26]. Microvariants were found at loci DYS627 (20.2), DYS458 (16.2), DYS19 (14.2), and DYS570 (21.3). All variants were confirmed by repeating the analysis.

### 3.2. Mutation Breakdown

**Figure 2:**
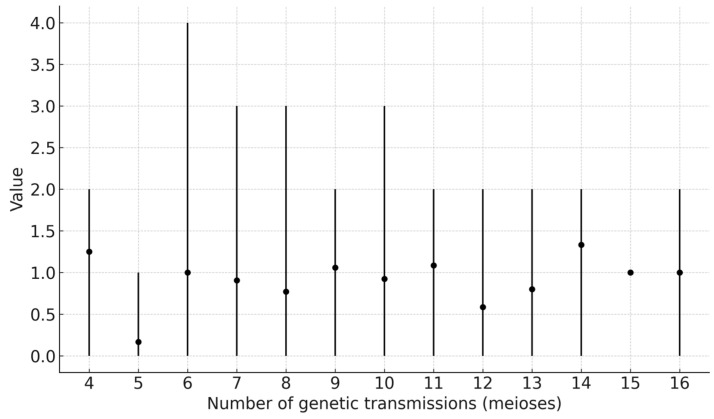
Average number of observed mutational counts across 4–16 genetic transmissions (meioses). Vertical whiskers denote the observed min–max range at each meiosis count.

We observed 161 mutations (Figure 2, Table S1), noting the assumptions outlined in Section 2.4. Most of the mutations were single-step mutations (n = 157, 97.5%), with a small proportion being deletions or duplications that were observed in one individual but not the other (n = 4, 2.5%). There were eight instances where multi-step differences between patrilineal individuals were interpreted as multiple single-step mutations, as they were not father–son pairs (in which case, a single multi-step event would have been accepted as the only valid scenario).

Duplications, deletions, and even triplications are well documented within the Y chromosome [27,28]. In addition to the four instances where a duplication or deletion was observed in one individual but not the other (shown in Table S1), we observed three duplications or deletions in our samples which were evident in both individuals of the pair, demonstrating that these allele patterns can be inherited (Table 1). For example, FS_2 and FS_269 displayed a duplication (alleles 15,17 and 15,16, respectively) at DYS19. DYS19 was the first forensically useful Y-STR and has since become ubiquitous in the fields of Y-chromosomal forensic analysis and evolutionary studies. Deletions, duplications, and triplications are well documented within this locus and are reported within the National Institute of Standards and Technology (NIST) STRBase [27,29,30,31].

For FS_375, a deletion was observed in DYS448. Both samples of the pair were re-amplified to confirm the deletion, and the remaining loci in the multiplex had sufficient signal, indicating this was not allelic dropout. However, we note that this may not represent a true deletion but instead a mutation in the primer binding site that prevented amplification. As we did not use alternative primers to verify this, the possibility of a null allele due to primer binding site mutation cannot be excluded. Deletions at DYS448 have also been reported in the NIST STRbase [32].

### 3.3. Mutation Rate

**Figure 3:**
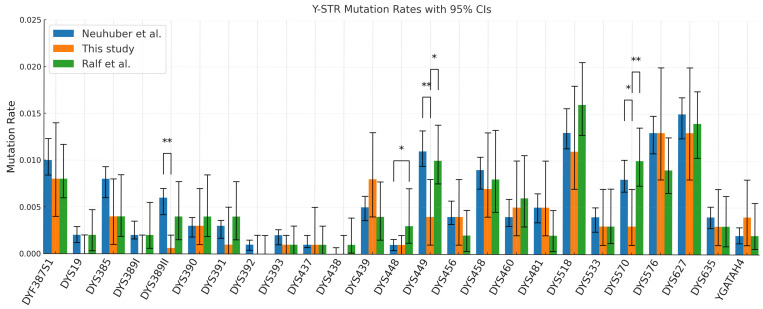
Mutation rate estimates for Yfiler Plus loci estimated from distantly related pairs separated by 4 to 16 meioses, n = 183 pairs. Our pedigree-derived estimates were compared against father–son estimates by Neuhuber et al. [15], as well as large-scale pedigree-based estimates by Ralf et al. [19]. * *p* < 0.05, ** *p* < 0.01.

Pedigree-based mutation rates were estimated and compared to mutation rate reference values which were derived from multiple father–son studies reported by Neuhuber et al. [15], as well as large-scale pedigree-based estimates by Ralf et al. [19] (Figure 3, Table 2). Notably, like Ralf et al. [19], we observed high concordance between mutation rates estimated from pedigree data and those based on father–son pairs obtained from Neuhuber et al. [15] (22 of the 25 Y-STRs analysed in our study, *p* > 0.05). Three of the markers showed a significant difference (*p* < 0.05) between the two ways of estimating rates: DYS389II, DYS449, and DYS570.

Like father–son estimates, pedigree-based estimates are not without limitations. For example, their reliability can be reduced by uncertainties arising from parallel mutations, back or forward mutations, and multi-step changes [5,21], and inaccuracies in reported biological relationships within pedigrees may introduce further error [20]. Despite these challenges, our findings showed that, for 22 of the 25 Y-STRs examined, the pedigree-based mutation rate estimates did not differ significantly from the father–son reference rates derived from large datasets. This high level of concordance indicates that pedigrees provide a valid and reliable framework for estimating Y-STR mutation rates.

Only three of the Y-STRs examined showed a significant difference compared to previous father–son rates. It is difficult to determine the exact cause of this; it could have been a result of the method employed to estimate the rate; the result of stochastic effects caused by the small sample size; or the result of the different biogeographic origin of the individuals included in the studies (because different populations exhibit different allelic distributions and therefore may exhibit higher or lower mutability for specific markers) [5,16]. Interestingly, though, for DYS449 and DYS570, our data also showed significant differences between our pedigree-based estimates and those by Ralf et al. [19], suggesting these are not the result of the method employed. Instead, these three loci may be outliers due to random sampling variation given the moderate number of events. Despite this, the strong agreement between the father–son and pedigree-based mutation rate estimates shown here indicates that pedigrees provide a valid framework for estimating mutation rates.

### 3.4. Male Differentiation Rate

The male relative differentiation rate for a given panel of Y-STRs refers to the rate at which a pair of paternally related males (such as brothers or first cousins) can be distinguished from one another by at least one allelic variation (mutation) across the markers. Across all 183 pairs separated by 4 to 16 meioses, 110 pairs exhibited ≥1 mutation, yielding an overall differentiation rate of 60.1%. This estimate is consistent with previous pedigree-based studies utilising the Yfiler Plus kit, where we observed a differentiation rate of 59.9% for pairs separated by 4 to 13 meiosis, compared with the 66.5% reported by Ralf et al. [19] for the same meiosis range. Among differentiated pairs, 97.3% differed at ≤2 loci and no pair exceeded four mutational differences. Although most pairs showed only a single mutation, forty pairs (21.9%) exhibited two, four pairs (2.2%) exhibited three, and one pair (0.5%) exhibited four. These results reflect the shallow accumulation of Y-STR changes across lineages, in line with observations from other deep pedigree studies [4,21,22,33], although this previous work did not employ Yfiler Plus.

Mutational events were not randomly distributed across loci. Instead, they were concentrated at RM Y-STRs, with DYS627 (38 pairs), DYS576 (35 pairs), DYF387S1 (33 pairs), and DYS518 (32 pairs) accounting for the majority of observed changes. This reinforces earlier findings that these loci, because of their elevated mutation rates, are especially discriminatory in patrilineal differentiation [4]. Our results therefore support previous conclusions that the incorporation of RM Y-STRs into Yfiler Plus substantially enhances the resolution of male relative differentiation (e.g., [10]).

### 3.5. Historical Remains Identification

Our data provide practical guardrails for interpreting Y-STR results when reference samples are available only from distant paternal relatives, as is common in historical human identification casework [23] and some missing-persons or mass-fatality contexts. Three features of our results are especially consequential: (i) the high overall male relative differentiation rate (~60% at 4–16 meioses), (ii) the concentration of mutational events at RM loci, and (iii) the shallow accumulation of mutational differences across many transmissions, with most differentiated pairs showing only one or two discordant loci and no pair showing more than four.

Our results indicate that distant patrilineal relatives can frequently differ at ≥1 Y-STR locus even when the relationship hypothesis is true. In our study, most differentiated true relatives diverged at only a small number of loci (typically ≤2), and multi-locus discordance >2 was rare. Operationally, this means that observing one to two discordant loci between a set of historical remains and a distant paternal reference should not be treated as definitive evidence against relatedness. Instead, the discordance must be weighed against (a) the number of separating meioses, (b) locus-specific mutation rates (especially for RM loci), and (c) plausible mutation pathways (including parallel and back mutations). Applying rigid ‘zero-mismatch’ expectations to distant relatives inflates the false-exclusion risk and is inconsistent with the empirical distribution we observed and with prior work using earlier panels (e.g., [4,22]).

Most observed changes arose at RM loci, reaffirming their contribution to both differentiation and interpretive complexity. When discordance is confined to one RM locus, particularly at high mutational markers, the evidential impact against relatedness is modest; by contrast, multiple independent differences at conventional (lower mutational) loci are more probative. Analysts should therefore adopt locus-aware weighting rather than treating any single mismatch as equivalent across the panel. Our concordance with father–son rate compilations for most loci (Figure 3) supports using those rates directly in likelihood calculations.

Current Scientific Working Group on DNA Analysis Methods interpretation guidelines [34] do not explicitly address scenarios where multiple discordant Y-STR haplotypes must be compared. In missing-person casework, it is common for several male relatives to be available; however, as shown here, their Y-STR haplotypes may differ because of mutational events (or misreported kinship). Rather than forcing the data into a single ‘consensus’ haplotype or discarding discordant references, casework should explicitly model alternative pedigree placements for each reference and evaluate the evidence jointly. Hence, more advanced interpretive approaches are needed, and likelihood-ratio methods have been proposed as a solution. Ge et al. [35], for example, extended the pedigree likelihood-ratio framework, already used with autosomal loci [36,37], to lineage markers such as Y-STRs and mitochondrial DNA. This method allows analysts to evaluate competing pedigree hypotheses, incorporate mutation rates, and express evidential weight as a likelihood ratio. While promising, such methods are not widely adopted, largely because of limited user-friendly software availability and the absence of detailed operational guidelines. Despite this, based on our data, we recommend moving away from binary ‘match/mismatch’ language toward quantitative likelihood ratios that condition on the proposed degree of relatedness (number of meioses); integrate locus-specific mutation rates; and allow for limited sets of discordances, especially at RM loci, under the relatedness hypothesis. The locus-specific mutation rates reported here can be incorporated into pedigree LR analyses following Ge et al. [35] by modelling a stepwise mutation process across *m* meioses under H1 (specified patrilineal relationship) and combining with calibrated haplotype probabilities under H2 (unrelated) that accommodate the population substructure (θ). A full LR case series requires population-matched databases and θ sensitivity analyses and is therefore beyond the scope of this rate estimation study.

## 4. Conclusions

Using 27 Yfiler Plus loci across 183 pedigrees (366 men) separated by 4–16 meioses, we show that pedigree-based estimation yields locus-specific mutation rates that largely agree with father–son datasets (22/25 loci), supporting pedigrees as a robust framework for rate estimation when direct transmissions are unavailable. Mutational changes were concentrated at RM loci (e.g., DYS627, DYS576, DYF387S1, DYS518), reaffirming their inflated contribution to patrilineal discrimination. The male differentiation rate observed in this study was 60.1%, with most differentiated pairs showing ≤2 locus differences and no pair showing more than 4. These dynamics are advantageous for excluding unrelated males within populations, yet they also highlight a key risk in historical or missing-persons casework, namely, that with increasing meioses, accumulated mutations can render true paternal relatives discordant at one or more loci. Accordingly, rigid mismatch thresholds (e.g., no more than three to five differences) are inappropriate for distant kinship inference and may lead to false exclusions. These findings reinforce the importance of contextual interpretation, especially during historical remains identification, and provide a foundation for improved forensic methodologies.

## Figures and Tables

**Table 1:** Inherited duplications or deletions.

Family ID	Sample 1	Sample 2	Meioses	Locus	Allele Sample 1	Allele Sample 2	Comments
FS_2	RS_13	RS_29	8	DYS19	15, 17	15, 17	Duplication mutation (gain/loss of 1 repeat), also observed at DYF387S1
FS_375	RS_942	RS_961	8	DYS448	NEG	NEG	Deletion mutation (duplication in one individual), also observed in DYF387S1
FS_269	RS_588	RS_574	5	DYS19	15, 16	15, 16	Duplication

**Table 2:** Locus-specific mutation rates for Yfiler Plus loci obtained from distantly related pairs separated by 4 to 16 meioses. Significant values are shown in bold. Confidence intervals were calculated using the Clopper–Pearson method. *p* values were calculated using Fisher’s exact method.

	Current Study					Neuhuber et al. [15]		Ralf et al. [19]	
Marker	Total Meioses	Mutations	Mutation Rate	Lower 95% CI	Upper 95% CI	Mutation Rate	*p* Value	Mutation Rate	*p* Value
DYF387S1	1576	13	0.008	0.004	0.014	0.010	0.587	0.008	1.000
DYS19	1576	0	0.000	0.000	0.002	0.002	0.102	0.002	0.255
DYS385	1576	6	0.004	0.001	0.008	0.008	0.109	0.004	1.000
DYS389I	1576	0	0.000	0.000	0.002	0.002	0.070	0.002	0.130
DYS389II	1576	1	0.001	0.000	0.002	0.006	**0.006**	0.004	0.077
DYS390	1576	4	0.003	0.001	0.007	0.003	1.000	0.004	0.564
DYS391	1576	2	0.001	0.000	0.005	0.003	0.575	0.004	0.193
DYS392	1576	0	0.000	0.000	0.002	0.001	0.611	0.000	1.000
DYS393	1576	1	0.001	0.000	0.002	0.002	0.503	0.001	1.000
DYS437	1576	2	0.001	0.000	0.005	0.001	1.000	0.001	0.597
DYS438	1576	0	0.000	0.000	0.002	0.000	1.000	0.001	0.503
DYS439	1576	12	0.008	0.004	0.013	0.005	0.135	0.004	0.166
DYS448	1576	1	0.001	0.000	0.002	0.001	1.000	0.003	0.134
DYS449	1576	6	0.004	0.001	0.008	0.011	**0.003**	0.010	**0.016**
DYS456	1576	6	0.004	0.001	0.008	0.004	1.000	0.002	0.317
DYS458	1576	11	0.007	0.004	0.013	0.009	0.658	0.008	0.844
DYS460	1576	8	0.005	0.002	0.010	0.004	0.678	0.006	0.820
DYS481	1576	8	0.005	0.002	0.010	0.005	0.842	0.002	0.126
DYS518	1576	18	0.011	0.007	0.018	0.013	0.636	0.016	0.185
DYS533	1576	5	0.003	0.001	0.007	0.004	1.000	0.003	1.000
DYS570	1576	5	0.003	0.001	0.007	0.008	**0.030**	0.010	**0.008**
DYS576	1576	20	0.013	0.008	0.020	0.013	1.000	0.009	0.241
DYS627	1576	21	0.013	0.008	0.020	0.015	0.822	0.014	1.000
DYS635	1576	5	0.003	0.001	0.007	0.004	0.828	0.003	1.000
YGATAH4	1576	6	0.004	0.001	0.008	0.002	0.140	0.002	0.528

## Data Availability

The datasets presented in this article are not readily available due to privacy and ethical restrictions.

## Supplementary Materials

The following supporting information can be downloaded at https://www.mdpi.com/article/10.3390/genes16101211/s1, Table S1: Mutations observed in 183 distantly related pairs.

## Abbreviations

The following abbreviations are used in this manuscript:

μ	Mutation rates
CI	Confidence interval
NIST	National Institute of Standards and Technology
RM	Rapidly mutating
UWC-A	Unrecovered War Casualties—Army
YHRD	Y Chromosome Haplotype Reference Database
Y-STRs	Y-chromosome short tandem repeats
