# Supplementary material for: Pedigree-Based Estimation of Y-STR Mutation and Male Differentiation Rates: Application to Historical Remains Identification
Source: Genes (Basel). 2025 Oct 14;16(10):1211. doi: 10.3390/genes16101211 (PMC12562835; doi:10.3390/genes16101211)
Supplement: Supplementary file 1 [file genes-16-01211-s001.zip › genes-3864741-supplementary.pdf]

Table S1. Mutations observed in 183 distantly related pairs.

Family ID	Sample 1	Sample 2	Meioses	Locus	Allele Sample 1	Allele Sample 2	Comments
FS_136	RS_114	RS_172	7	DYF387S1	37,40	36,39	Mutation gain/loss of 1 repeat)
FS_246	RS_624	RS_495	8	DYF387S1	36,36	36,37	Mutation (gain/loss of 1 repeat)
FS_249	RS_1414	RS_498	7	DYF387S1	36,37	37,37	Mutation (gain/loss of 1 repeat)
FS_542	RS_1331	RS_1310	8	DYF387S1	36,36	35,36	Mutation (gain/loss of 1 repeat)
FS_2	RS_13	RS_29	8	DYF387S1	38,39	39,40	Mutation (gain/loss of 1 repeat)
FS_375	RS_942	RS_961	8	DYF387S1	38,39	39	Mutation (duplication in one individual)
FS_475	RS_1116	RS_1115	9	DYF387S1; DYS456	35,36,37; 15	35,37; 16	Mutation (duplication in one individual); Mutation (gain/loss of 1 repeat)
FS_463	RS_1290	RS_1362	8	DYF387S1; DYS458	35,37; 18	36,37; 17	Mutation (gain/loss of 1 repeat)
FS_118	RS_133	RS_279	6	DYF387S1; DYS458; DYS576	38,38; 13; 16	37,38; 14; 17	Mutation (gain/loss of 1 repeat)
FS_311	RS_708	RS_788	10	DYF387S1; DYS460	35,36; 11	36,36; 12	Mutation (gain/loss of 1 repeat)
FS_344	RS_1210	RS_776	7	DYF387S1; DYS627	35,36; 24	35,37; 23	Mutation (gain/loss of 1 repeat)
FS_338	RS_754	RS_849	10	DYS385; DYS627	9,14; 22	11,14; 23	Mutation (gain/loss of 1 repeat); Mutation (gain/loss of 2 repeats)
FS_17	RS_137	RS_212	10	DYS390; DYS576	22; 18	23; 19	Mutation (gain/loss of 1 repeat)
FS_514	RS_1276	RS_1291	11	DYS390; DYS627	26; 26	25; 25	Mutation (gain/loss of 1 repeat)
FS_146	RS_263	RS_334	6	DYS437; DYS449; DYS456; DYS576	15; 28; 16; 18	14; 29; 15; 19	Mutation (gain/loss of 1 repeat)
FS_262	RS_488	RS_525	6	DYS439; DYS518	13; 41	12; 40	Mutation (gain/loss of 1 repeat)
FS_374	RS_1391	RS_960	6	DYS439; DYS518	11; 37	12; 38	Mutation (gain/loss of 1 repeat)
FS_442	RS_1053	RS_1003	8	DYS439; DYS518; DYS576	13; 38; 18	12; 39; 17	Mutation (gain/loss of 1 repeat)
FS_265	RS_593	RS_605	6	DYS439; DYS576	12; 19	11; 18	Mutation (gain/loss of 1 repeat)
FS_494	RS_1205	RS_1202	7	DYS439; DYS627	12; 24	13; 23	Mutation (gain/loss of 1 repeat)
FS_30	RS_354	RS_353	7	DYS449	32	33	Mutation (gain/loss of 1 repeat)
FS_385	RS_1097	RS_1080	7	DYS449	31	32	Mutation (gain/loss of 1 repeat)
FS_95	RS_99	RS_317	12	DYS449; DYS458	30; 17	31; 18	Mutation (gain/loss of 1 repeat)

FS_395	RS_1091	RS_1017	11	DYS449; DYS518	31; 36,37	30; 37	Mutation (gain/loss of 1 repeat); Mutation (duplication in one individual)
FS_158	RS_233	RS_221	6	DYS449; DYS635	28; 22	29; 21	Mutation (gain/loss of 1 repeat)
FS_304	RS_612	RS_656	8	DYS458; DYS518	16; 38	17; 39	Mutation (gain/loss of 1 repeat)
FS_332	RS_785	RS_856	13	DYS458; DYS576	15; 17	16; 18	Mutation (gain/loss of 1 repeat)
FS_470	RS_1453	RS_1454	16	DYS458; DYS627	19; 22	18; 21	Mutation (gain/loss of 1 repeat)
FS_506	RS_1266	RS_1260	9	DYS458; DYS627	18; 24	17; 23	Mutation (gain/loss of 1 repeat)
FS_223	RS_413	RS_571	10	DYS481; DYS576	22; 20	21; 19	Mutation (gain/loss of 1 repeat)
FS_126	RS_205	RS_659	6	DYS518	39	40	Mutation (gain/loss of 1 repeat)
FS_160	RS_366	RS_494	7	DYS518	38	37	Mutation (gain/loss of 1 repeat)
FS_268	RS_644	RS_563	8	DYS518	38	39	Mutation (gain/loss of 1 repeat)
FS_346	RS_783	RS_816	10	DYS518	40	39	Mutation (gain/loss of 1 repeat)
FS_430	RS_1234	RS_1226	8	DYS518	42	43	Mutation (gain/loss of 1 repeat)
FS_530	RS_1337	RS_1319	8	DYS518	39	38	Mutation (gain/loss of 1 repeat)
FS_552	RS_1405	RS_1409	9	DYS518; DYS570	39; 22	38; 21	Mutation (gain/loss of 1 repeat)
FS_121	RS_303	RS_132	14	DYS518; DYS576	39; 18	40; 17	Mutation (gain/loss of 1 repeat)
FS_24	RS_438	RS_826	8	DYS518; DYS576	35; 16	36; 17	Mutation (gain/loss of 1 repeat)
FS_366	RS_927	RS_963	8	DYS518; DYS576	39; 17	38; 18	Mutation (gain/loss of 1 repeat)
FS_83	RS_863	RS_917	9	DYS518; DYS576	38; 20	39; 21	Mutation (gain/loss of 1 repeat)
FS_422	RS_1128	RS_1162	7	DYS518; DYS627; YGATAH4	43; 15; 13	42; 16; 12	Mutation (gain/loss of 1 repeat)
FS_208	RS_1196	RS_567	8	DYS518; YGATAH4	39; 12	38; 13	Mutation (gain/loss of 1 repeat)
FS_313	RS_770	RS_916	6	DYS570	17	18	Mutation (gain/loss of 1 repeat)
FS_473	RS_1403	RS_1449	11	DYS570	19	18	Mutation (gain/loss of 1 repeat)
FS_533	RS_1316	RS_1338	7	DYS570	25	24	Mutation (gain/loss of 1 repeat)
FS_193	RS_419	RS_852	9	DYS570; DYS627	17; 22	18; 21	Mutation (gain/loss of 1 repeat)
FS_115	RS_157	RS_322	8	DYS576	17	18	Mutation (gain/loss of 1 repeat)
FS_308	RS_667	RS_690	10	DYS576	17	18	Mutation (gain/loss of 1 repeat)
FS_339	RS_738	RS_742	12	DYS576	17	18	Mutation (gain/loss of 1 repeat)
FS_391	RS_903	RS_899	8	DYS576	20	18	Mutation (gain/loss of 2 repeats)
FS_408	RS_1095	RS_1024	10	DYS576	18	20	Mutation (gain/loss of 2 repeats)
FS_389	RS_893	RS_909	10	DYS576; DYS627	17; 24	18; 25	Mutation (gain/loss of 1 repeat)

FS_537	RS_1364	RS_1450	11	DYS576; DYS627	19; 24	18; 25	Mutation (gain/loss of 1 repeat)
FS_109	RS_348	RS_602	15	DYS627	20	21	Mutation (gain/loss of 1 repeat)
FS_114	RS_208	RS_431	8	DYS627	23	22	Mutation (gain/loss of 1 repeat)
FS_185	RS_880	RS_283	6	DYS627	23	22	Mutation (gain/loss of 1 repeat)
FS_221	RS_343	RS_474	11	DYS627	22	23	Mutation (gain/loss of 1 repeat)
FS_316	RS_790	RS_703	9	DYS627	22	23	Mutation (gain/loss of 1 repeat)
FS_372	RS_1151	RS_933	7	DYS627	23	24	Mutation (gain/loss of 1 repeat)
FS_467	RS_1140	RS_1180	8	DYS627	23	24	Mutation (gain/loss of 1 repeat)
FS_489	RS_1186	RS_1213	12	DYS627	21	20	Mutation (gain/loss of 1 repeat)
FS_491	RS_1223	RS_1208	8	DYS627	23	22	Mutation (gain/loss of 1 repeat)
FS_77	RS_706	RS_868	9	DYS627	18	19	Mutation (gain/loss of 1 repeat)
FS_196	RS_363	RS_422	6	DYS627; YGATAH4	23; 12	24; 13	Mutation (gain/loss of 1 repeat)
FS_149	RS_225	RS_878	10	DYS385	11,15	11,14	Mutation (gain/loss of 1 repeat)
FS_517	RS_1351	RS_1307	11	DYS385	14,15	14,14	Mutation (gain/loss of 1 repeat)
FS_462	RS_1110	RS_1114	9	DYS385; DYS458	15,16; 17	15,15; 16	Mutation (gain/loss of 1 repeat)
FS_12	RS_235	RS_213	14	DYS385; DYS533	13,17; 12	13,18; 13	Mutation (gain/loss of 1 repeat)
FS_225	RS_769	RS_845	6	DYS389II	31	32	Mutation (gain/loss of 1 repeat)
FS_480	RS_1157	RS_1149	8	DYS390	23	24	Mutation (gain/loss of 1 repeat)
FS_521	RS_1322	RS_1384	9	DYS390	23	24	Mutation (gain/loss of 1 repeat)
FS_26	RS_758	RS_733	12	DYS391	12	11	Mutation (gain/loss of 1 repeat)
FS_288	RS_540	RS_774	8	DYS391	10	11	Mutation (gain/loss of 1 repeat)
FS_498	RS_1250	RS_1238	8	DYS393	13	12	Mutation (gain/loss of 1 repeat)
FS_390	RS_904	RS_885	11	DYS437; DYS458	16; 15	15; 16	Mutation (gain/loss of 1 repeat)
FS_139	RS_166	RS_190	6	DYS439	11	12	Mutation (gain/loss of 1 repeat)
FS_450	RS_1019	RS_1015	8	DYS439	11	13	Mutation (gain/loss of 2 repeats)
FS_481	RS_1192	RS_1348	11	DYS439	11	12	Mutation (gain/loss of 1 repeat)
FS_92	RS_94	RS_57	6	DYS439	14	13	Mutation (gain/loss of 1 repeat)
FS_327	RS_697	RS_721	9	DYS439; DYS481	14; 24	15; 23	Mutation (gain/loss of 1 repeat)
FS_66	RS_43	RS_261	4	DYS439; YGATAH4	12,13; 12	12; 11	Mutation (duplication in one individual); Mutation (gain/loss of 1 repeat)
FS_27	RS_325	RS_79	10	DYS448; DYS533	20; 13	19; 12	Mutation (gain/loss of 1 repeat)
FS_150	RS_1365	RS_1398	10	DYS456	16	15	Mutation (gain/loss of 1 repeat)

FS_319	RS_922	RS_749	10	DYS456	16	15	Mutation (gain/loss of 1 repeat)
FS_4	RS_20	RS_52	6	DYS456	18	17	Mutation (gain/loss of 1 repeat)
FS_485	RS_1168	RS_1259	12	DYS456	16	15	Mutation (gain/loss of 1 repeat)
FS_133	RS_178	RS_465	11	DYS458	16	17	Mutation (gain/loss of 1 repeat)
FS_310	RS_677	RS_684	10	DYS458	18	19	Mutation (gain/loss of 1 repeat)
FS_103	RS_226	RS_832	7	DYS460	12	11	Mutation (gain/loss of 1 repeat)
FS_144	RS_179	RS_312	7	DYS460	10	11	Mutation (gain/loss of 1 repeat)
FS_162	RS_243	RS_232	8	DYS460	12	11	Mutation (gain/loss of 1 repeat)
FS_226	RS_674	RS_981	7	DYS460	11	10	Mutation (gain/loss of 1 repeat)
FS_388	RS_901	RS_905	8	DYS460	10	11	Mutation (gain/loss of 1 repeat)
FS_65	RS_55	RS_45	5	DYS460	11	12	Mutation (gain/loss of 1 repeat)
FS_90	RS_158	RS_105	7	DYS460	12	11	Mutation (gain/loss of 1 repeat)
FS_209	RS_368	RS_515	6	DYS481	26	27	Mutation (gain/loss of 1 repeat)
FS_266	RS_1217	RS_635	10	DYS481	22	23	Mutation (gain/loss of 1 repeat)
FS_276	RS_580	RS_611	8	DYS481	28	27	Mutation (gain/loss of 1 repeat)
FS_283	RS_681	RS_746	12	DYS481	22	23	Mutation (gain/loss of 1 repeat)
FS_482	RS_1209	RS_1199	10	DYS481	24	25	Mutation (gain/loss of 1 repeat)
FS_55	RS_32	RS_822	4	DYS481	24	23	Mutation (gain/loss of 1 repeat)
FS_219	RS_516	RS_875	6	DYS533	13	12	Mutation (gain/loss of 1 repeat)
FS_477	RS_1148	RS_1221	13	DYS533	11	12	Mutation (gain/loss of 1 repeat)
FS_287	RS_506	RS_508	4	DYS533; DYS635	12; 23	13; 22	Mutation (gain/loss of 1 repeat)
FS_155	RS_180	RS_202	13	DYS635	25	26	Mutation (gain/loss of 1 repeat)
FS_272	RS_686	RS_1074	7	DYS635	24	25	Mutation (gain/loss of 1 repeat)
FS_317	RS_886	RS_956	8	DYS635	21	22	Mutation (gain/loss of 1 repeat)
FS_260	RS_501	RS_864	9	YGATAH4	12	13	Mutation (gain/loss of 1 repeat)
FS_296	RS_912	RS_578	8	YGATAH4	13	12	Mutation (gain/loss of 1 repeat)
